# 

**Published:** 1889-01-26

**Authors:** 


**Notice to Advertisers and Subscribers.**

All Advertisements, Orders for Copies of THE HOSPITAL, and Business Communications should be addressed to The Publisher, *not to the Editor*, THE HOSPITAL, 38, Old Jewry, E.C.

**To Residents, Secretaries, and Matrons.**

The Editor will be glad to have the Regulations and each Report as issued by Asylums, Hospitals, Convalescent and Nursing Institutions, as well as those of other Charities, and an early copy in duplicate of all Appeals and Festival Notices, etc., addressed to *The Editor, The Lodge, Porchester Square, London, W.* Any superintendent, secretary, matron, or other chief official who regularly sends such information may be registered, on application to the Publisher, to receive free of cost a cover for binding THE HOSPITAL in half-yearly volumes.

A Scale of Charges will be found on the first page of the NURSING SUPPLEMENT.

### SCRAPS AND GLEANINGS.

THE Swansea Hospital Ball took place on January 8th.

LINCOLN COUNTY HOSPITAL has invested £6,000 at 3½ per cent.

THE Galen Club will probably be merged into the Grosvenor Club.

THE Empress Frederick has visited the German Hospital at Dalston.

THE death is announced of Professor A. Pagenstecher, of Heidelberg University.

THE students of Middlesex Hospital gave a dance at the College on January 23rd.

A YOUNG lady walked over the cliffs of Cardiff last week in a fit of somnambulism.

MR. SANDERSON is giving a series of concerts at Monkwearmouth in aid of the dispensary.

THE Bath Eastern Dispensary ends a year of good work with a balance in hand of nearly £100.

THE workpeople of Armitage and Rigby Mills have subscribed £16 to the Warrington Infirmary.

LAST Sunday was the 19th anniversary of Hospital Sunday in Liverpool, and was kept as such.

DR. BAILEY has been elected house surgeon of Stockport Infirmary, in place of Dr. Greenhalgh resigned.

THERE is a comic song, called "The Dispensary Doctor," which we recommend for hospital concerts.

MR. JOHN ELWIN'S entertainment at Brompton Hospital, on January 15th, was, as usual, a great success.

A MEETING will be held at Forres on January 23rd, to elect the governors of the new college hospital.

AN anonymous benefactor is going to plant with pine the grounds of Dublin Royal Hospital for Incurables.

A VERY pleasant entertainment was given to the patients of Great Yarmouth Hospital on December 29th.

ACCOMMODATION for private patients of Montrose Asylum will be supplied in the houses of Dr. Howden and Mr. Lyall.

THE meetings of the Leeds Workpeople's Hospital Funds were held last week in the Mill-hill, West, and North-West Wards.

A SPECIAL meeting was held on January 16th to consider the serious position of Longton Cottage Hospital, which is in debt £171.

THE Prince of Wales has been obliged through press of engagements to decline the invitation to open the Bath Hospital, Harrowgate.

M. PASTEUR has resigned his post of perpetual secretary of the Academy of Sciences, on account of the bad state of his health.

A MEETING will be held at the Mansion House on January 29th to consider Mr. Coote's penny scheme for aiding the London hospitals.

HER Majesty the Queen has been pleased to forward a quantity of old linen for use in the wards of the Cancer Hospital (Free), Brompton.

AT the annual meeting of the Hull Infirmary Convalescent Institution, on January 14th, an urgent appeal was made for further subscriptions.

THE sanitary improvements at Cumberland Infirmary are now finished. £910 has been spent on drainage work, and £540 for heating apparatus.

MISS WOOD gave a lengthy address to the nurses of Salop Infirmary on January 3rd, pressing them to join the British Nurses' Association.

### AMUSEMENTS AND RELAXATION.

### NEW PUZZLE PRIZE SCHEME.

SECOND QUARTERLY COMPETITION COMMENCED JANUARY 5TH, ENDS MARCH 30TH, 1889.

1. Prizes will be awarded for the best Original Puzzles in Prose or Verse relating to any of the Social, Political, or Philanthropical topics of the day. Competitors are requested to *strictly* observe this rule, or their contributions will be discredited.
2. Puzzles may take any form which the ingenuity of the competitors suggest. Answers must accompany contributions, and the source of obscure or ambiguous terms be given.
3. No competitor will be allowed to take more than one prize during the Quarter.
4. Eight Prizes of Standard Books will be given, the choice of the book to be left to the winner. First, Second, Third, Fourth, and Fifth Prizes for best Original Puzzles, £1 1s., 15s., 10s. 6d., 7s. 6d., and 5s. each.
5. First, Second, and Third Prize for Solutions of 15s., 10s. 6d., and 5s. each.

N.B.—All contributions must reach the office, 38, Old Jewry, E.C., not later than the *First Post* on *Thursday* in each week.

### SPECIAL NOTICE.

N.B.—The Prize Editor regrets that more competitors have not entered for the present quarter. Doubtless the holidays may have something to do with this, and he trusts that all intending competitors will forward their contributions without further delay; and that they, with those who have already entered the lists, will call the attention of their puzzle-making friends to this column of the paper, which, from its retired position, is unknown to many of the numerous readers of THE HOSPITAL.

### PUZZLES FOR SOLUTION.

**Fourth Week.**

No. 8. DOUBLE ACROSTIC.

My first agrees with ready skill,
To help and cheer my last;
My latter works my former's will,
Although the task is vast.

1. Sparkling with a thousand rays,
A sign which kingly power displays.
2. Lightly its strains float o'er the ear,
To lovers of music entrancing dear.
3. This with its number is often the test,
Dividing the baser from that which is best.
4. How many have mounted my summit in fear,
As the enemy's forces were drawing near.
5. He whose deeds of jealous rage,
Hath oft been pictured on the stage.
6. Delivery decides my fate,
As first, second, or other rate.

PATIENCE.

No. 9. TRIPLE ACROSTIC.

My centrals at the present time
With first and last agree;
Though ere long, in this changing clime,
Both first and last may flee.

1. A Scotch drink.
2. A class of reptiles.
3. A formidable animal.
4. A determined aim.

TINIE.

**Marks for Original Puzzles.**

| Names. | No. of Puzzles sent in Jan. 17. | No. of Marks Jan.17. | Totals. | Names. | No. of Puzzles sent in Jan. 17. | No. of Marks Jan.17. | Totals. |
|---|---|---|---|---|---|---|---|
| Cotonopolis... | 3 | 5 | 12 | Padre ......... | — | — | 3 |
| Tinie........... | 3 | 5 | 11 | Prior........... | 3 | 2 | 4 |
| Jenny Wren . | — | — | 4 | Lightowlers . | 1 | 2 | 2 |
| Patience ...... | 3 | 5 | 11 | "Twentieth" | 1 | 3 | 3 |

**Answers.**

No. 4. DOUBLE ACROSTIC.

C lam P
H armonic A
R egio N
I nstrumen T
S trappad O
T er M
M aor I
A lu M
S pit E

No. 5. THE A B C OF "THE HOSPITAL ANNUAL."

A. *Annual.*
B. *Burdett* broached it.
C. *Charity* called for it.
D. *Duty* dictated it.
E. *England* examined it.
F. *Falsehood* fled from it.
G. *Governors* gloried in it.
H. *Hospitals* hailed it.
I. *Institutions* inquired for it.
J. *Justice* joyed in it.
K. *Knowledge* kindled it.
L. *Liberality* listened to it.
M. *Magazines* magnified it.
N. *Nurses* noted it.
O. *Officers* ordered it.
P. *Proprietors* published it.
Q. *Questions* quickened it.
R. *Reform* required it.
S. *Science* subscribed to it.
T. *Truth* taught it.
U. *Understanding* undertook it.
V. *Vestries* valued it.
W. *Weakness* welcomed it.
X. *Xlence* xemplified it.
Y. *You* yearned for it.
Z. *Zeal* zested it.

**Marks for Solution of Puzzles.**

Total Marks.—Flora (0), 1; Mater, 0; Scrooge (0), 1; Patience 0; Lady Clara (0), 2; Ruffles (0), 1; Lauriston, 0; Jenny Wren, 0.

(For Conditions, see last week's THE HOSPITAL.)

